# Trophic Structure and Mercury Biomagnification in Tropical Fish Assemblages, Iténez River, Bolivia

**DOI:** 10.1371/journal.pone.0065054

**Published:** 2013-05-31

**Authors:** Marc Pouilly, Danny Rejas, Tamara Pérez, Jean-Louis Duprey, Carlos I. Molina, Cédric Hubas, Jean-Remy D. Guimarães

**Affiliations:** 1 Institut de Recherche pour le Développement - UMR Borea- Biologie des Organismes et des Ecosystèmes Aquatiques (MNHN, CNRS, IRD, UPMC), Paris, France; 2 Unidad de Limnología y Recursos Acuáticos, Universidad Mayor de San Simón, Cochabamba, Bolivia; 3 Institut de Recherche pour le Développement – US Imago, La Paz, Bolivia; 4 Instituto de Ecología, Unidad de Limnología, Universidad Mayor de San Andrés, La Paz, Bolivia; 5 Muséum National d’Histoire Naturelle - UMR Borea MNHN, CNRS, IRD, UPMC, Paris, France; 6 Universidade Federal do Rio de Janeiro, Inst. de Biofisica Carlos Chagas Filho, Lab. de Traçadores, Rio de Janeiro, Brazil; Ecole Normale Supérieure de Lyon, France

## Abstract

We examined mercury concentrations in three fish assemblages to estimate biomagnification rates in the Iténez main river, affected by anthropogenic activities, and two unperturbed rivers from the Iténez basin, Bolivian Amazon. Rivers presented low to moderate water mercury concentrations (from 1.25 ng L^−1^ to 2.96 ng L^−1^) and natural differences in terms of sediment load. Mercury biomagnification rates were confronted to trophic structure depicted by carbon and nitrogen stable isotopes composition (δ^15^N; δ^13^C) of primary trophic sources, invertebrates and fishes. Results showed a slight fish contamination in the Iténez River compared to the unperturbed rivers, with higher mercury concentrations in piscivore species (0.15 µg g^−1^ vs. 0.11 µg g^−1^ in the unperturbed rivers) and a higher biomagnification rate. Trophic structure analysis showed that the higher biomagnification rate in the Iténez River could not be attributed to a longer food chain. Nevertheless, it revealed for the Iténez River a higher contribution of periphyton to the diet of the primary consumers fish species; and more negative δ^13^C values for primary trophic sources, invertebrates and fishes that could indicate a higher contribution of methanotrophic bacteria. These two factors may enhance methylation and methyl mercury transfer in the food web and thus, alternatively or complementarily to the impact of the anthropogenic activities, may explain mercury differences observed in fishes from the Iténez River in comparison to the two other rivers.

## Introduction

Mercury, and its organic form methyl mercury, that is easily assimilated and accumulated in aquatic food chains, constitute a major environmental and public health issue in the Amazonian context. Mercury inputs may originate from exogenous sources related to gold mining or industrial uses, but also come from natural sources of mercury accumulated and trapped in the soils along the geological history of the basin [1]. This endogen mercury is liberated by natural or anthropogenic erosions and transported by lixiviation towards the aquatic systems. Contamination is thereafter controlled by a set of biotic and abiotic conditions among which methylation rates [2]–[4] and amplification processes along the food chain [5], [6] are key factors. Food uptake represents more than 85% of the methylmercury total uptake, well above passive uptake from water [7], and amplification processes along the food chain may increase the mercury concentration several thousand fold from water to fish top predators. Two major amplification processes, bioaccumulation and biomagnification, are likely to control mercury concentrations in organisms [5]. Bioaccumulation refers to the increase of mercury concentrations along the lifetime of an individual while biomagnification is defined as the increment of mercury concentration between the successive consumer levels of the food chain. Biomagnification is assumed to be positively linked to food chain length, that may be derived from Nitrogen stable isotope analysis [8], [9]. Food source origin and pathway could also be determinant: sediment biofilm, phytoplankton and periphyton are potential food sources and also support mercury methylation [2], [3], [10] in relation to the activity of sulfate-reducing [11] and methanogen [12] bacteria.

A previous study concluded that mercury concentration in fishes from the Iténez could not be completely explained by bioaccumulation processes [13]. In this study, we examined mercury concentrations in a fish assemblage to compare biomagnification rates in three rivers from the Iténez basin with low to moderate water mercury concentrations (from 1.25 ng L^−1^ to 2.96 ng L^−1^). They also differ in their natural sediment load (clear vs. white waters) and anthropogenic activities (deforestation and gold mining activity). We hypothesized that these differences are likely to affect biological production, food web structure and consequently mercury biomagnification rates. Accordingly, stable carbon and nitrogen isotopic composition (δ^13^C; δ^15^N) were measured in trophic sources, invertebrates and fish in order to evaluate the relationship between biomagnification rates, food web sources and trophic chain length.

## Methods

### Ethic Statement

ULRA/UMSS laboratory is an Authorized Scientific Institution (ICA) accredited by the Bolivian Dirección General de la Biodiversidad y Áreas Protegidas (DGBAP, Viceministerio de Medio Ambiente, Ministerio de Medio Ambiente y Agua) to conduct biological scientific research within the Bolivian territory, including protected areas (Resolución administrativa BMABCC 026/09). IRD is linked to ULRA/UMSS through cooperation agreements.

This particular project has been approved and permissions for biological collects have been issued by DGBAP, departmental Prefecture of Beni, Iténez departmental park (PD-AMNI Iténez) and local authorities (Remanso, Mategua, Versalles and Bella Vista villages).

Local fishermen captured and manipulated fish according to procedures permitted by the Viceministerio de Medio Ambiente. Rapidly after the capture, living fishes were manually sacrificed (by percussive stunning) or left in high doses of anaesthetic (phenoxy-ethanol) to minimize suffering. Local fish assemblage did not involve endangered or protected species.

### Study Area

**Figure 1:**
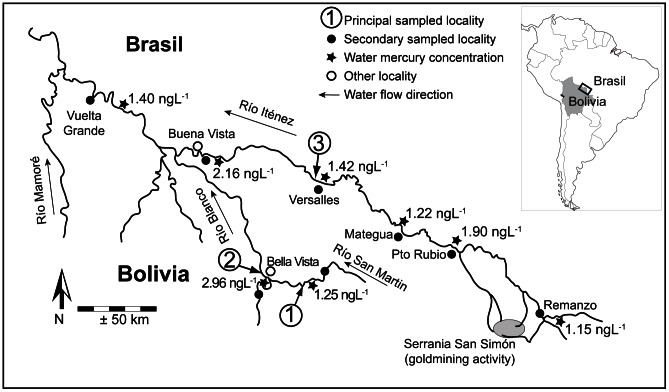
Hydrological map of the Bolivian part of the Iténez basin. Sampling locations (black point) and water mercury concentrations (star) are indicated. The principal sampled locations were: 1- San Martín River; 2- Blanco River; 3- Iténez River. (Redrawn from [13]).

The study was carried out in three rivers of the Iténez basin: San Martín River, Blanco River and the main Iténez River (Figure 1, see [13] for further details on the basin, rivers and studied sites). They present differences in river water chemistry mainly related to their sediment load and mercury concentration in water. Iténez and San Martín rivers present clear, yellow to green waters characteristic of low sediment load (mean suspended particulate matter concentration [SPM] of 7.3 and 11.4 mg L^−1^, respectively [13]). On the contrary, Blanco River drains white waters with higher sediment load ([SPM] of 26.1 mg L^−1^). Iténez River is affected by deforestation in the Brazilian territory and by a gold mine (Serranía San Simón, Bolivia). Blanco and San Martín rivers belong to the same catchment, mainly covered by tropical forest. They present low human population densities and globally low anthropogenic impact. Flooding area and duration are likely to be higher in the Iténez main river. Satellite mapping of flood and vegetation (based on SAR and J-ERS images, see method in [14]) indicated flooding areas of 15–20% for the San Martin basin, 20–25% for Blanco basin and 30–35% for the main Iténez river basin in its central part [15]. Mercury shows high affinity with sediment particles and, for that reason, its total concentration in water ([Hg]) increases with sediment load [16], [17]. As a consequence, waters from the Blanco River and its floodplain lakes naturally present higher [Hg] (mean [Hg] = 2.96 ng L^−1^ in river; 1.52 ng L^−1^ in lakes) than those from the Iténez (1.54 ng L^−1^; 1.26 ng L^−1^) and San Martín rivers (1.25 ng L^−1^; 0.64 ng L^−1^), ([13], Figure 1). All these mercury concentrations are low compared with the regional Amazonian context ([Hg]_total_ from 1 to 35 ng L^−1^ [18]). However, higher values observed in the Iténez River, compared to the other clear water San Martín River, suggest that this system is slightly perturbed [13].

Different floodplain lakes of each river were visited but most of the samples were collected in two lakes of each river (Figure 1): Curicha (12°36′48″ S–63°26′11″ W) and Negra (12°37′48″ S–63°24′40″ W) in the Iténez River; Cambarazal (13°17′58″ S–63°36′37″ W) and Redonda (13°18′16″S–63°33′14″ W) in the San Martín River; Zacarias (13°15′47″ S–63°42′39″ W) and La Granja (13°16′04″ S–63°42′56″ W) in the Blanco River. Other samples (<15%) were collected in secondary localities to complete the data set (see positions on Figure 1). Some fishes (<10%) were collected during two previous field trips (November 2004 and August 2005) but most of the fish and all the trophic source samples were collected during the dry season of 2007 in three dates in the Iténez River (June, September and November), and six dates in the Blanco and San Martín River (monthly from June to November).

All the studied lakes were located near the river mainstream (between 50 and 200 m) so that they received water from the river during the high water season but remained isolated during the dry season. This ensured that fish sampled during the dry season had been living in the fishing site at least during the precedent two or three months.

### Material

Material from potential fish food sources were collected in floodplain lakes in order to evaluate their isotopic signatures: terrestrial plants (tree leaves from the lake bank), C3 (*Eichhornia crassipes, Pistia stratioides, Polygonum sp. and Cyperus sp.)* and C4 (*Paspalum repens*) aquatic macrophytes, periphyton (epiphytic biofilm), particulate organic matter (POM, obtained by successive water filtration onto a 20-µm mesh and a pre-combusted glass fibre GF/F filter), leaf litter (mainly decaying leaves of terrestrial plants collected from the bottom of the lakes) and common groups of aquatic macroinvertebrates (Odonata, Decapoda, Ephemeroptera, Coleoptera and Gasteropoda). Samples were rinsed with ultra-pure (milli-Q) water, stored in individual tubes or bags, and stored frozen until their analysis.

Fishes were captured with gill nets (2.5 m height × 25 m long, mesh sizes of 20, 25, 30, 35, 40 and 50 mm). We collected specifically fishes of eight species and four trophic levels to represent the fish assemblage: Detritivore/algivore: *Curimatella cf. alburna* and *Psectrogaster* sp.; Herbivore: *Schizodon fasciatus*; Microcarnivore (insectivore): *Triportheus angulatus*; Generalist piscivore: *Pseudoplatystoma* sp. and *Pygocentrus* nattereri; Specialized piscivore: *Acestrorhynchus* sp. and *Hoplias malabaricus*.

Fishes were identified and measured (Standard Length, SL in cm) and 4–5 g of dorsal muscle tissue were extracted using an ultra clean sampling procedure [19] and taking care to exclude blood, skin or bones. All the fish muscle samples were frozen in individual tubes. Size ranges of studied individuals were set to include only adults, less subject to dietary shifts, and to obtain comparable size ranges between the three populations studied for each species.

In the laboratory, samples were lyophilized to obtain a completely dry extract, and grounded to a fine powder to perform mercury and isotopic analysis.

### Mercury Analyses

The Laboratorio de Calidad Ambiental (LCA) from Instituto de Ecología of La Paz University (Bolivia) carried out mercury analyses on fish muscle samples. Mercury was extracted by acid digestion and quantified by cold vapour atomic fluorescence spectroscopy (CVAFS, Brooks Rand Model III see [13] for further details on the protocol). Results were expressed as total mercury concentration in wet weight muscle ([Hg]_ww_ in µg g^−1^). A previous work showed that some populations present a significant influence of fish size on mercury concentration [13]. So fish size was selected to be similar between populations and limited to adult range and then [Hg] values were not corrected by fish size.

### Isotopic Analysis

Nitrogen (δ^15^N) and carbon (δ^ 13^C) stable isotope ratios of food sources, invertebrates and fishes were measured to describe food web structure in the three locations studied. δ^15^N was used to estimate consumer trophic position as consumers are constantly δ^15^N enriched in comparison to their preferred food source; on the contrary, the δ^13^C is relatively stable among trophic levels but varies in relation with the sources that support the food chain and rather indicates energy pathway [20].

Relative individual trophic position (TP) was calculated by the formula: TP = λ+(δ^15^N_fish_−δ^15^N_base_)/Δ (where λ is the trophic position of the organism used to estimate δ^15^N_base_ and Δ is the N isotopic fractionation in ‰ that occurs between each trophic level). The isotopic fractionation value Δ was set to 2.8‰ [21]. δ^15^N_base_ was estimated using mean δ^15^N of the detritivore species *C. alburna* and then λ was set to 2. UC Davis Stable Isotope Facility laboratory (University of California, Davis, USA, http://stableisotopefacility.ucdavis.edu/) performed the isotopic analyses.

### Statistical Analysis

In order to evaluate differences in isotopic signatures 1) between source categories, 2) between species, 3) between the three rivers for each species and source categories and to test differences in mercury concentration between species, we employed Kruskal-Wallis (K–W, non parametric Anova) and Permanova tests (permutational multivariate Anova that may consider simultaneously the δ^13^C and δ^15^N values; available on the Vegan package of the R statistical computing freeware program http://www.r-project.org/, [22]). Homogeneity of multivariate dispersion was tested with a permutation test prior to Permanova.

Relative contributions of primary food sources to isotopic signature of primary consumer fish species (detritivore and herbivore) were estimated applying a Bayesian mixing model (SIAR R-package [23]) in order to depict differences in river food web source that may explain differences in biomagnification. This model allows to estimate probability distributions of multiple source contributions to an isotopic signature while accounting for the observed variability in source, mixture isotopic signatures and isotopic fractionation [23]. Nevertheless the selection of a small set of sources is required to provide a better resolution of the results [24]. Stomach contents information (based on qualitative field trip observation and [25]) was used to depict large diet categories of fish species and to select the sources.

A biomagnification factor was calculated as the ratio between the maximum and minimum species [Hg] mean values. This factor was completed by the evaluation of the slope of the TP vs. [Hg] relation (Log transformed). Finally, a relative food chain length was evaluated for each river by mean trophic level of the four piscivore species. Differences of food chain length values between rivers were tested by Kruskal-Wallis. For all tests, type I error was set to p = 0.05.

## Results

### Trophic Structure

**Figure 2:**
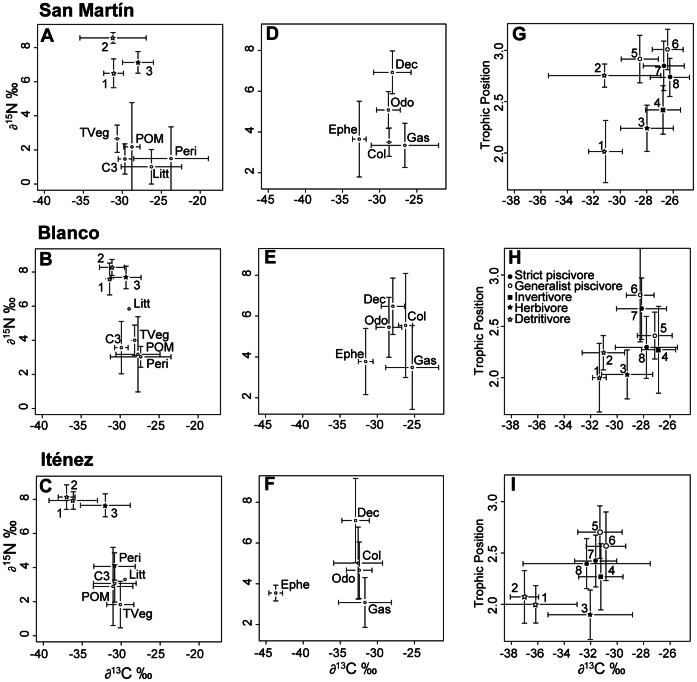
Isotopic signature of sources, invertebrates and fish in three rivers of the Iténez basin (Amazon, Bolivia). Biplots display mean values (± sd in error bars) of δ^13^C and δ^15^N values for sources (**A,B,C**) POM =  Particulate organic matter, Litt =  Leaf litter, Peri =  Periphyton; C3 =  C3 aquatic macrophytes and TVeg =  Terrestrial Vegetation; and invertebrates groups (**D,E,F**): Ephe =  Ephemeroptera, Odo =  Odonata, Dec =  Decapoda, Col =  Coleoptera and Gas =  Gasteropoda. (**G,H,I**) represented mean values (± sd in error bars) of δ^13^C and Trophic Position (derived from δ^15^N) of fish species: detritivore *(1 =  Curimatella cf. alburna, 2 =  Psectrogaster* sp.), herbivore *(3 =  Schizodon fasciatus)*, insectivore (4 = Triportheus angulatus) and piscivore (5 = *Pseudoplatystoma* sp., 6 = *Pygocentrus* nattereri, 7 = *Hoplias malabaricus and 8 = Acestrorhynchus* sp.). In biplots (**A,B,C**), C4 aquatic marcophytes (δ^13^C : −12.3 to −13.2‰; δ^15^N : 2 to 2.2‰) were not plotted and δ^13^C and δ^15^N values of detritivore (1, 2) and herbivore (3) fish species were reported.

Isotopic signatures of primary food sources were significantly different (Permanova, p = 0.001) between the six categories (terrestrial plants, C3 and C4 macrophytes, periphyton, leaf litter and POM); but differences became non significant (Permanova, p = 0.075) when excluding the C4 macrophytes that presented the highest δ^13^C values (varying between −13.2‰ and −12.3‰) in comparison to the other food source categories that oscillated between −35‰ and −25‰ (Table 1). These five categories were not significantly different among them for δ^13^C values (Kruskal-Wallis, K–W, p = 0.064) nor for δ^15^N values (K–W, p = 0.056). Periphyton (Permanova, p = 0.002) and POM (Permanova, p = 0.012) isotopic signatures presented significant variation between localities, being more ^13^C depleted and ^15^N enriched in the Iténez River in comparison to Blanco and San Martín rivers (Table 1, Figure 2a,b,c). The remaining sources presented no significant differences (Permanova, p>0.05) in δ^13^C and δ^15^N values.

**Table 1:** Isotopic composition (δ15N, δ13C) of food sources and invertebrates in three rivers of the Iténez basin.

	River	n	δ^15^N (‰)				δ^13^C (‰)			
			mean	sd	min	max	mean	sd	min	max
Source										
Periphyton	Blanco	15	3.03	0.61	1.49	3.75	−27.36	3.89	−29.62	−13.71
	Iténez	24	4.07	0.80	2.83	5.76	−30.86	2.66	−35.30	−22.30
	San Martin	9	1.50	1.86	−1.03	4.25	−23.75	4.79	−26.95	−13.31
POM	Blanco	15	3.18	2.20	−0.85	7.38	−27.71	2.83	−32.43	−23.12
	Iténez	9	2.89	2.29	−1.52	5.01	−31.04	2.54	−34.75	−28.15
	San Martin	10	2.18	2.59	−2.05	5.80	−28.77	1.04	−30.08	−27.47
C3 macrophytes	Blanco	4	3.57	1.54	2.54	5.83	−29.85	0.87	−30.67	−29.06
	Iténez	16	3.07	1.10	1.85	5.51	−30.82	2.74	−36.24	−26.40
	San Martin	4	1.47	0.89	0.92	2.78	−29.67	0.86	−30.92	−29.08
C4 macrophytes	Blanco	0	–	–	–	–	–	–	–	–
	Iténez	4	1.95	1.78	0.64	4.58	−13.24	0.73	−14.00	−12.53
	San Martin	1	2.15	–	–	–	−12.28	–	–	–
Terrestrial plants	Blanco	2	4.00	0.89	3.37	4.63	−28.14	0.37	−28.40	−27.88
	Iténez	13	1.83	1.37	0.01	4.83	−30.11	1.74	−34.06	−27.34
	San Martin	2	2.66	0.80	2.09	3.22	−30.66	0.33	−30.89	−30.43
Leaf litter	Blanco	1	5.84	–	–	–	−28.85	–	–	–
	Iténez	1	3.30	–	–	–	−29.51	–	–	–
	San Martin	3	1.01	1.01	0.12	2.11	−26.26	3.88	−29.55	−21.98
Invertebrate										
Coleoptera	Blanco	2	3.50	0.69	3.01	3.99	−28.73	0.31	−28.95	−28.51
	Iténez	7	5.02	1.77	2.75	6.97	−32.59	3.29	−38.63	−28.33
	San Martin	2	5.54	2.56	3.74	7.34	−26.21	0.94	−26.87	−25.54
Decapods	Blanco	8	6.92	1.05	4.76	7.95	−28.30	2.52	−31.28	−24.32
	Iténez	18	7.10	2.07	2.27	9.92	−32.94	1.85	−35.52	−29.32
	San Martin	5	6.48	1.38	4.95	8.54	−27.91	1.56	−29.54	−26.06
Ephemeroptera	Blanco	4	3.65	1.86	2.18	6.28	−32.75	0.92	−33.59	−31.46
	Iténez	4	3.55	0.39	3.03	3.89	−43.69	0.93	−44.99	−42.94
	San Martin	3	3.77	1.62	2.73	5.64	−31.56	0.99	−32.6	−30.63
Gasteropods	Blanco	3	3.34	1.08	2.12	4.18	−26.61	4.54	−29.94	−21.44
	Iténez	6	3.08	1.22	1.79	4.66	−31.67	3.57	−35.02	−26.86
	San Martin	3	3.48	2.05	1.98	5.82	−25.31	3.55	−28.85	−21.75
Odonates	Blanco	7	5.08	0.90	4	6.23	−28.83	1.59	−31.86	−26.97
	Iténez	11	4.67	1.39	1.69	5.89	−32.45	1.73	−34.24	−28.29
	San Martin	8	5.45	1.47	3.55	7.95	−28.44	1.73	−31.13	−26.44

n =  sample number.

Isotopic signatures of the five invertebrate groups (Odonata, Decapoda, Ephemeroptera, Coleoptera and Gasteropoda) were significantly different among them (Permanova, p = 0.001; Table 1). Differences between groups for the δ^13^C values (K–W, p = 0.0158) concerned principally the Ephemeroptera that were ^13^C depleted (δ^ 13^C from −45‰ to −33‰) compared to the other groups (δ^ 13^C oscillating between −36‰ and −27‰). Ephemeroptera and Gasteropoda showed the lowest δ^15^N values (population means between 3.08‰ and 3.77‰), Coleoptera and Odonata were intermediate (3.5‰ – 5.54‰) and Decapoda showed the highest values (6.48‰–7.1‰). Isotopic compositions between the three rivers were significantly different for the Decapoda, Ephemeroptera and Odonata (Permanova, p = 0.001, 0.003 and 0.002, respectively) but not significantly different for Coleoptera and Gasteropoda (Permanova, p = 0.053 and p = 0.092, respectively). For the first three groups δ^13^C values were significantly lower in the Iténez River in relation to the other rivers (K–W, p = 0.0001, 0.015 and 0.001, respectively, Figure 2d,e,f), although δ^15^N values were not significantly different between rivers (K–W, p>0.5). Carbon isotope ratios of Coleoptera and Gasteropoda tended to be ^13^C depleted in the Iténez River as well (Table 1, Figure 2d,e,f).

For the Iténez River, all the invertebrate groups presented more negative δ^13^C values (from −43.69 to −31.67‰) than primary food sources (−31.04 to −29.51‰, Table 1, Figure 2d,e,f).

All the three rivers merged, significant differences in the isotopic signature between fish species were found (Permanova, p = 0.001) and species were gradually positioned on the trophic position axis in accordance to their coarse diet regime (Figure 2g,h,i). The eight fish species also showed significant differences among rivers (Permanova, p = 0.001, Table 2). Trophic position (TP) of piscivore species varied significantly between rivers (K–W, p<0.005) and was higher in the San Martín River (between 2.7 and 3) than in the two other sites (between 2.3 and 2.7). On the contrary, non-piscivore species did not present significant differences (K–W, p>0.2), except for *Psectrogaster sp.* (K–W, p = 0.01) that also showed a higher trophic level in the San Martín River.

**Table 2:** Standard Length, mercury concentration and isotope signature (δ15N and relative Trophic Position - TP, δ13C) of eight fish species populations sampled in three rivers of the Iténez basin (Amazon, Bolivia).

		Standard Length (mm)			[Hg]ww (µg g^−1^)				δ^ 15^N (‰)		TP		δ^ 13^C (‰)	
Species	River	n*	mean	sd	n*	mean	sd	n*	mean	sd	mean	sd	mean	sd
*Curimatella cf alburna*	Blanco	8	144.0	15.1	8	0.07	0.06	8	7.60	0.94	2.00	0.33	−31.35	0.52
	Iténez	19	150.1	9.6	18	0.05	0.03	19	7.93	0.51	2.00	0.18	−36.15	3.11
	San Martin	19	153.9	11.0	18	0.04	0.02	19	6.49	0.85	2.00	0.30	−31.11	1.27
*Psectrogaster sp.*	Blanco	8	132.6	19.7	4	0.07	0.02	4	8.28	0.47	2.24	0.17	−31.05	1.60
	Iténez	49	132.3	16.5	49	0.06	0.02	31	8.13	0.72	2.07	0.26	−37.01	1.08
	San Martin	8	156.5	35.1	7	0.04	0.02	3	8.57	0.31	2.74	0.11	−31.18	4.25
*Schizodon fasciatus*	Blanco	30	235.3	41.0	23	0.04	0.02	26	7.68	0.67	2.03	0.24	−29.25	1.93
	Iténez	65	211.3	44.6	59	0.05	0.03	59	7.65	0.67	1.90	0.24	−32.04	3.19
	San Martin	38	239.7	25.6	22	0.04	0.02	35	7.13	0.63	2.23	0.23	−27.98	1.99
*Triportheus angulatus*	Blanco	30	137.0	18.7	21	0.07	0.04	27	8.36	0.18	2.27	0.42	−26.87	1.29
	Iténez	44	143.5	19.9	38	0.08	0.04	35	8.68	0.90	2.27	0.32	−31.24	1.67
	San Martin	23	155.4	32.4	18	0.07	0.04	19	7.63	0.66	2.41	0.24	−26.77	1.32
*Pseudoplatystoma sp.*	Blanco	6	420.7	25.5	6	0.13	0.10	6	8.74	0.63	2.41	0.23	−27.16	1.31
	Iténez	60	512.2	163.4	58	0.15	0.08	47	9.90	0.71	2.70	0.26	−31.30	1.68
	San Martin	13	438.7	74.5	13	0.17	0.10	7	9.02	0.64	2.90	0.23	−28.52	1.40
*Pygocentrus nattereri*	Blanco	26	187.8	32.4	20	0.14	0.09	25	9.85	0.27	2.80	0.45	−28.26	1.05
	Iténez	96	177.8	52.2	76	0.19	0.10	94	9.52	0.94	2.57	0.33	−30.84	1.49
	San Martin	32	200.0	27.8	28	0.10	0.05	25	9.28	0.55	2.99	0.20	−26.42	1.17
*Acestrorhynchus sp.*	Blanco	15	171.3	33.7	14	0.07	0.03	15	8.42	0.89	2.30	0.30	−27.80	2.35
	Iténez	53	189.5	33.5	49	0.12	0.07	51	9.04	0.68	2.40	0.24	−32.88	1.45
	San Martin	31	202.3	35.0	23	0.10	0.06	27	8.52	0.52	2.72	0.19	−26.26	1.48
*Hoplias malabaricus*	Blanco	36	280.8	71.1	24	0.09	0.04	32	9.48	0.84	2.67	0.30	−28.16	1.89
	Iténez	74	250.0	58.7	65	0.13	0.07	67	9.11	0.71	2.42	0.25	−31.62	1.59
	San Martin	38	309.0	61.2	26	0.09	0.09	32	8.83	0.68	2.84	0.24	−26.72	1.54

* n = fish (sample) number. Differences exist on fish numbers because isotopic and mercury analyses were not always performed on all the individuals.

As for periphyton, POM and invertebrates, fish species globally tended to be more ^13^C depleted in the Iténez River (Figure 2). Fish assemblage values ranged between −31‰ and −26‰ in San Martín River, −33‰ and −27‰ in Blanco River and −37‰ and −31‰ in Iténez River. These differences persisted and were significant for all species (K–W, p<0.0005). In the Iténez River, as for the invertebrates, the two detritivore species (*Psectrogaster* sp. and *C. alburna*) also presented more negative δ^13^C than all the considered food sources (Figure 2a,b,c).

Relative contribution of primary food sources to detritivore fish species may be biased because these species presented more negative δ^13^C than all the considered food sources; this was not the case for the herbivore *S. fasciatus* (Table 3). However, the three species showed a similar pattern, with a high contribution of periphyton in the Iténez River (68–80%) and low contribution (2–16%) in the two other rivers. The contribution of terrestrial vegetation followed a reverse pattern, being lower in Iténez River (2–3%) than in the two remaining rivers (18–79%). The contribution of terrestrial vegetation was the highest for *S. fasciatus* and C. *alburna* in San Martín River (67 and 79%, respectively). No dominant primary food source category appeared in the diet of the three species in the Blanco River.

**Table 3:** Source relative contributions (mean % ± sd, estimated by SIAR mixing model) to detritivore (*Psectrogaster* sp. and *Curimatella cf. alburna*) and herbivore (*Schizodon fasciatus*) fish diet in three rivers of the Iténez basin.

River/Species	Peri (%)	POM (%)	C3 (%)	C4 (%)	TVeg (%)	Litt (%)
Blanco						
*Psectrogaster sp.*	14±10	15±10	18±11	6±6	19±11	28±12
*Curimatella cf. alburna*	15±10	17±10	23±12	4±4	18±11	23±9
*Schizodon fasciatus*	12±8	10±7	31±10	1±1	18±11	29±7
Itenez						
*Psectrogaster sp.*	80±10	5±5	6±6	1±1	2±2	6±6
*Curimatella cf. alburna*	68±14	8±8	9±8	2±2	3±3	10±8
*Schizodon fasciatus*	79±6	5±4	7±5	1±1	2±2	6±5
San Martin						
*Psectrogaster sp.*	16±10	18±10	17±10	13±9	19±10	16±10
*Curimatella cf. alburna*	2±2	8±7	7±6	1±1	79±10	3±2
*Schizodon fasciatus*	4±4	16±13	5±5	5±2	67±13	3±3

Peri = Periphyton; POM = Particulate organic matter; C3 =  C3 aquatic macrophytes; C4 =  C4 aquatic macrophytes; TVeg = Terrestrial vegetation; Litt =  Leaf litter.

Relative food chain length, evaluated by mean trophic level of the four piscivore species, presented significant differences (K–W, p<0.0001) with higher values in San Martín River (2.86) in comparison to Blanco River (2.55) and in the Iténez River (2.52).

### Fish Mercury Concentration and Biomagnification

**Figure 3:**
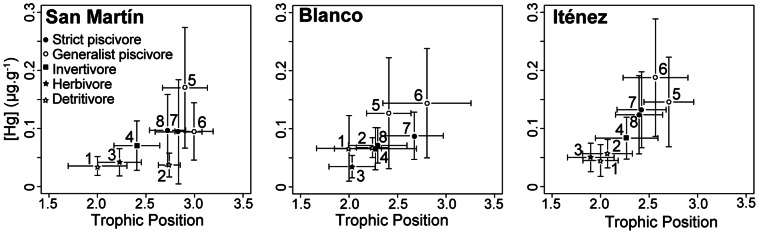
Mercury biomagnification in fish assemblage of three rivers from the Iténez basin (Amazon, Bolivia). Biplots display mean values (± sd in error bars) of Trophic Position (derived from δ^15^N) and [Hg]_ww_ of detritivore *(1 = Curimatella cf. alburna, 2 = Psectrogaster* sp.), herbivore *(3 = Schizodon fasciatus)*, insectivore (4 = Triportheus angulatus) and piscivore (5 = *Pseudoplatystoma* sp., 6 = *Pygocentrus* nattereri, 7 = *Hoplias malabaricus and 8 = Acestrorhynchus* sp.) fish species. Slope of the relation correspond to biomagnification along the food chain.

Fish species presented significant differences in mercury concentrations (K–W, p<0.0001) that could be related to their coarse diet regime in agreement with biomagnification processes (Figure 3). At the assemblage level we found a significant global correlation (Spearman *ρ* = 0.579, p<0.0001) between individual mercury concentrations and trophic position that was still valid individually for each river (San Martín: *ρ* = 0.678; Blanco: *ρ* = 0.633 and Iténez: *ρ* = 0.654, all p<0.0001).

Piscivore species showed significantly higher mercury concentrations in the Iténez River (0.151 µg g^−1^±0.08, n = 248) than in San Martin and Blanco rivers (0.106 µg g^−1^±0.08, n = 90 and 0.105 µg g^−1^±0.07, n = 64 respectively) (K–W, p<0.0001). A similar difference (K–W, p = 0.005) also occurred for detritivore and herbivore species with higher values in the Iténez River (0.052 µg g^−1^±0.03, n = 126) than in the two others (0.046 µg g^−1^±0.03, n = 35 in Blanco River and 0.039 µg g^−1^±0.02, n = 47 in San Martin River). At the species level, four species showed significant differences in mercury concentrations between rivers (K–W: *Acestrorhynchus* sp., p = 0.006; *H. malabaricus,* p = 0.0001; *P. nattereri,* p<0.0001 and *S. fasciatus,* p = 0.024), all of them presented higher values in the Iténez River (Table 2).

Biomagnification factor, calculated as the ratio between [Hg] of *P. nattereri* (species with the highest mean [Hg] = 0.16 µg g^−1^) and *C. alburna* (lowest mean [Hg] = 0.05 µg g^−1^), was 2.5 in the San Martín, 2 in the Blanco and 3.8 in the Iténez River. Similarly, the slope of the relationship between δ^15^N and [Hg] (Log transformed) was higher in Iténez River (slope = 0.43, R^2^ = 0.82, p<0.001) than in the Blanco River (slope = 0.34, R^2^ = 0.70, p = 0.02) and in the San Martín River where the relation was not significant (slope = 0.22, R^2^ = 0.45, p = 0.07) (Figure 3).

## Discussion

The three studied rivers presented a similar general pattern of food source contribution that is in agreement with knowledge from previous studies in the Amazon [26]–[30]. In particular, the isotopic signature of C4-macrophytes is clearly ^13^C enriched compared to the other primary sources and consumers, thus they are not a significant food source for consumers and can not sustain the food chains in the study sites. On the other hand, the other food sources may all contribute to the food web, but remained widely overlapped. However, although the three rivers are submitted to the same climatic conditions and belong to the same hydrographical basin, major differences in carbon isotopic signatures and food chain length could be detected:

1. Iténez River differed from the two others mainly because primary sources, primary consumers and secondary consumers were all more ^13^C depleted than in San Martín and Blanco rivers (Figure 2);
2. Iténez River also presented a higher contribution of periphyton to the diet of the detritivore and herbivore fishes (Table 3);
3. San Martín River showed a longer food chain than the two other rivers because of the higher trophic position of all piscivore species (Figure 2g,h,i), while the three rivers presented similar δ^15^N values for the five primary source categories considered (Table 1).

We hypothesized that natural variations of water quality (clear water with low sediment load vs. white water with high sediment load) would have an effect on trophic structure, as shown for instance in Venezuelan rivers [21]. In such a case, the two clear water rivers (Iténez and San Martin) would have shown a similar trophic structure and origin, and different from the one of the white water Blanco River. The results did not follow this pattern: Iténez River presented different carbon isotopic signature and periphyton contribution than the two other rivers; whereas, San Martin River showed a longer food chain in comparison to Iténez and Blanco rivers. It thus appears that sediment load was not a dominant factor controlling trophic structure in the lakes studied.

The more negative δ^13^C values for primary producers, invertebrates and fish from the Iténez River compared to those from the two other rivers indicate differences in carbon sources between rivers. Moreover, fish δ^13^C values, especially those of the detritivore species *C. alburna* (−36.1‰ in Iténez River) and *Psectrogaster* sp. (−37‰), as well as Ephemeroptera mayfly (−43.7‰), were more ^13^C depleted than the sampled primary producers (−29.5‰ to −31‰). The low positive isotopic fractionation of carbon (±1‰) that occurs between each trophic level [20] could not explain this discrepancy, that then implies the contribution of an additional (not sampled) ^13^C-depleted carbon source. Detritivore fish species and Ephemeroptera are likely to feed predominantly on the bottom near the sediment (see [31] for a discussion on Ephemeroptera feeding). Methane production from anoxic sediments could provide such ^13^C-depleted carbon source [32], [33]. Several studies demonstrated that methane-oxidizing bacteria (MOB) activity allows the transfer of this ^13^C-depleted carbon to zooplankton [34] and fish [35]. Thus, more ^13^C-depleted carbon could be an indicator of a contribution of methane carbon to benthic as well as pelagic lake food webs in temperate [36] and tropical [35] systems. Amazonian lakes and reservoirs can support a high methane production [37] and several studies observed low δ^13^C values in fish from South American tropical systems [28], [35], [38], [39]. In the Ichilo River (Bolivian Amazonian lowlands) Rejas [28] observed low δ^13^C values for algivore (*Anodus elongatus*, −39‰ ±0.3) and detritivore fishes (*Potamorhina altamazonica,* −36.4‰ ±1.2; *Psectrogaster rutiloides* −35.3‰ ±1.2) and even lower values for benthic invertebrates (Chironomidae, Ephemeroptera, −39.7‰ ±1.2) than for the most ^13^C depleted primary food source (POM, −37‰ ±0.6). Wantzen *et al.* [38] suggested that seasonal variations in methane production, induced by water level in the Brazilian Pantanal, might explain lower δ^13^C values during the wet season for the detritivore fish *Psectrogaster curviventris;* and Sanseverino *et al.* [35] demonstrated that the ^13^C signature of fishes is related to MOB activity. Lower δ^13^C values for invertebrates and fish in Iténez River than in the other rivers could then be tentatively interpreted as an effect of higher carbon production by metanotrophic bacteria. However, Molina *et al.* [31] did not report such low values in the Beni River (Bolivian Amazonian lowlands) where *Campsurus mayfly (Ephemeroptera)* presented similar δ^13^C values (−35.7 to −34.7‰) to seston (−35.1 to −33.8‰), revealing that this process is not a generality.

The three studied rivers presented relatively low water mercury concentration, similar to mercury levels found in natural systems of the region [13]. Due to their lower sediment load, clear water rivers, like Iténez and San Martin, should have demonstrated a naturally lower mercury concentration in comparison to Blanco River. Previous results [13] and this study showed a slightly perturbed situation in the Iténez River, with higher mercury concentrations in piscivore and herbivore species, compared to fish from non-perturbed rivers (Blanco and San Martín).

Based on a partially similar data set and sampling locations, Pouilly *et al.* [13] concluded that bioaccumulation, defined as the increment of mercury concentrations during an organism’s lifetime, is not the principal factor explaining increased mercury concentrations in fish from Iténez River. Conversely, Iténez River showed higher biomagnification factor (3.8) than the two other rivers (Blanco = 2, San Martín = 2.5), indicating that this process may partially explain higher mercury concentrations in fish from the Iténez River. We hypothesized that the trophic structure and in particular food chain length could control the biomagnification rate, because freshwater systems generally demonstrate a positive relationship between mercury biomagnification rates and food chain length [9], [30], [40]. However, the two clear water rivers studied showed an opposite relationship (Figure 3), with higher mercury biomagnification factor (3.8) and shorter food chain (2.52) in Iténez River, and lower biomagnification factor (2.5) longer food chain in (2.86) in the San Martín River. This discrepancy between the general pattern and the situation in the two studied clear water rivers could originate from a higher mercury bioavailability and/or a better efficiency in the transfer along the food web in the Iténez River. It has been suggested that periphyton and macrophytes constitute the main pathway of mercury between primary producers and macro-invertebrates in Canadian temperate lakes [41]. A strong link between methanogenic bacteria and mercury methylation in the periphyton has been demonstrated [12] and Dominique *et al.* [39] related the high methyl mercury concentrations found in detritivore fishes downstreams of a dam in French Guyana, to the export of methyl mercury from the reservoir and to the quality of the biofilm which is characterized by low δ13C values, indicating MOB activity. In the Amazonian systems, periphyton associated to macrophyte roots is a major mercury methylation site [3], [42] and higher biomagnification rates for invertebrates feeding on periphyton has been demonstrated [30]. In our study, estimation of food source contribution by the mixing model showed that the contribution of periphyton to the diet of the detritivore and herbivore fishes was high in the Iténez River and low in San Martín and Blanco rivers, and that a higher contribution of terrestrial vegetation, in particular for *S. fasciatus* and *C. alburna* in the San Martín River. A scheme of higher methylation rates due to methanogenic bacteria activity within biofilms (as indicated by the more negative δ^13^C values observed) and higher contribution of periphyton in the food web may explain the higher biomagnification rates observed in fish from the Iténez River in comparison to the two other rivers. Balance of internal (periphyton, phytoplankton) vs. external (terrestrial vegetation) primary production as well as MOB activity may thus be critical factors in food web mercury contamination.

The three rivers differ in their flooding regime, the main Iténez River showing larger flooding area and longer flooding duration, therefore more lake connectivity, than Blanco and San Martín rivers [15]. Apart from this difference, it remains unclear which other factors could generate a higher MOB activity and periphyton contribution in the Iténez River.

The observations reported correspond mainly to the 2007 dry season and a generalisation based on several years of studied would be necessary. For this date we can conclude that, in the Iténez basin with low to moderate mercury concentrations in water, fish mercury contamination appeared mainly controlled by biomagnification enhanced by periphyton contribution to food web and probably environmental conditions, such as flooding, favourable to methylation and methanogenesis. Surprisingly in these systems biomagnification rates were not related to food chain length, but rather to a methanogenic pathway. Our results also suggest that biomagnification, favoured by trophic structure and biotic processes, may lead to critical contamination of fishes even at low rates of mercury input.
